# Structural equation modelling exploration of the key pathophysiological processes involved in cardiac surgery-related acute kidney injury in infants

**DOI:** 10.1186/s13054-016-1350-1

**Published:** 2016-06-05

**Authors:** Mirela Bojan, Maria Constanza Basto Duarte, Natalia Ermak, Vanessa Lopez-Lopez, Agnès Mogenet, Marc Froissart

**Affiliations:** Department of Anesthesiology and Critical Care, Necker - Enfants Malades University Hospital, Assistance Publique - Hôpitaux de Paris, 149, rue de Sèvres, 75015 Paris, France; Anestesiologia Cardiovascular, Fundacion Cardiovascular de Colombia, Bucaramanga, Colombia; Department of Biochemistry, Necker-Enfants Malades University Hospital, Assistance Publique - Hôpitaux de Paris, Paris, France; Unité de Recherche Clinique, CIC Centre Necker Cochin, Assistance Publique - Hôpitaux de Paris, Paris, France; Inserm Unit 1018, CESP, Research Centre in Epidemiology and Population Health, Renal and Cardiovascular Epidemiology Team, Villejuif, France

**Keywords:** Paediatric cardiac surgery, Acute kidney injury, Cardiopulmonary bypass

## Abstract

**Background:**

Uncertainties about the pathophysiological processes resulting in cardiac surgery-related acute kidney injury (AKI) in infants concern the relative impact of the most prominent risk factors, the clinical relevance of changes in glomerular filtration rate vs tubular injury, and the usefulness of available diagnostic tools. Structural equation modelling could allow for the assessment of these complex relationships.

**Methods:**

A structural model was specified using data from a prospective observational cohort of 200 patients <1 year of age undergoing cardiopulmonary bypass surgery. It included four latent variables: AKI, modelled as a construct of perioperative creatinine variation, of oliguria and of urine neutrophil gelatinase-associated lipocalin (uNGAL) concentrations; the cardiopulmonary bypass characteristics; the occurrence of a post-operative low cardiac output syndrome and the post-operative outcome.

**Results:**

The model showed a good fit, and all path coefficients were statistically significant. The bypass was the most prominent risk factor, with a path coefficient of 0.820 (95 % CI 0.527–0.979), translating to a 67.2 % explanation for the risk of AKI. A strong relationships was found between AKI and early uNGAL excretion, and between AKI and the post-operative outcome, with path coefficients of 0.611 (95 % CI 0.347–0.777) and 0.741 (95 % CI 0.610–0.988), respectively. The path coefficient between AKI and a >50 % increase in serum creatinine was smaller, with a path coefficient of 0.443 (95 % CI 0.273–0.596), and was intermediate for oliguria, defined as urine output <0.5 ml kg^−1^ h^−1^, with a path coefficient of 0.495 (95 % CI 0.250–0.864). A path coefficient of −0.229 (95 % CI −0.319 to 0.060) suggested that the risk of AKI during the first year of life did not increase with younger age at surgery.

**Conclusions:**

These findings suggest that cardiac surgery-related AKI in infants is a translation of tubular injury, predominately driven by the cardiopulmonary bypass, and linked to early uNGAL excretion and to post-operative outcome.

**Trial registration:**

ClinicalTrials.gov identifier NCT01219998. Registered 11 October 2010.

**Electronic supplementary material:**

The online version of this article (doi:10.1186/s13054-016-1350-1) contains supplementary material, which is available to authorized users.

## Background

Acute kidney injury (AKI) is a common complication of cardiac surgery, and the occurrence of AKI is an independent risk factor for death following cardiac surgery in infants [1, 2]. Although almost all of the known risk factors for AKI in infants relate to impaired renal perfusion, the weight of each causal factor is still debated. Data in adults support a lower risk of AKI when the cardiac surgery (i.e., coronary artery bypass) is performed off-pump, incriminating cardiopulmonary bypass (CPB) itself as a trigger [3]. The design of previous studies in infants [2, 4–8] did not allow for the prioritization between the CPB-related and other pre-, intra- and post-operative risk factors of AKI.

A second subject of ongoing debate is the early diagnosis of AKI in infants undergoing cardiac surgery. Serum creatinine concentration (sCr) is acknowledged to be an inadequate marker for early diagnosis. This is because of compensatory mechanisms in the setting of pre-renal hypoperfusion, combined with slow variation of sCr after injury. sCr is not only slow to increase but often decreases in very young patients owing to the dilutional effects of CPB priming and post-operative fluid overload [9, 10]. Urine neutrophil gelatinase-associated lipocalin (uNGAL) is a marker of tubular injury which has been shown to reveal AKI before the rise in creatinine in infants [11–13]. However, tubular injury may not always couple with reductions in the glomerular filtration rate (GFR), and, conversely, reductions in GFR from pre-renal azotaemia may not always combine with tubular injury. A further unresolved issue is that of which pathophysiological process is more likely to be clinically relevant for AKI, and thus more important to monitor: changes in GFR or tubular injury. Focusing on the relationships between AKI and casual exposures, clinical patterns, concurrent testing and prognosis may help elucidate the consequences of attributing post-operative AKI to changes in tubular integrity instead of glomerular function, and improve the understanding of the overall spectrum of the disease [14].

Structural equation modelling (SEM) [15, 16] is a way to assess complex and multivariate relationships and can be used to test conceptual models. It also allows for the use of latent variables (i.e., variables which cannot be measured directly but which can be expressed by measurable ones). It is an appealing way to resolve diagnostic problems in the absence of an acknowledged diagnostic gold standard. The aim of the present work was to identify the main drivers leading to AKI in infants undergoing cardiac surgery, the clinical relevance of changes in GFR vs tubular injury, and the usefulness of available diagnostic tools. To do so, we investigated the complex relationships between AKI (modelled as a latent variable), observed causal exposures, clinical patterns, traditional biomarkers of AKI, uNGAL and outcome.

## Methods

### Study setting, design and participants

The study was conducted at the Necker-Enfants Malades University Hospital, Paris, France, after approval by the regional ethics committee, Paris Descartes University, France, and was performed with the financial support of the Direction de la Recherche Clinique, Assistance Publique - Hôpitaux de Paris, France. The ethics committee waived the need for written consent because data were anonymised through a deidentification process, and since samples consisted of urine collected through a urinary catheter that would have otherwise been discarded. As advised by the ethics committee, verbal consent was collected from all the participants’ parents. The study is registered with ClinicalTrials.gov (NCT01219998).

This observational study included 75 neonates (≤28 days old) and 125 infants undergoing cardiac surgery with CPB who were enrolled prospectively to explore the predictive ability of post-operative uNGAL concentrations for AKI. No pre-term infant was included. Surgery was performed with either normothermic, non-pulsatile CPB or deep hypothermic circulatory arrest (when reconstruction of the aortic arch was required), and surgical complexity was accounted for by using the Society of Thoracic Surgeons and European Association for Cardio-Thoracic Surgery Congenital Heart Surgery Mortality Score [17]. All patients were provided with standard care and monitoring according to the institution’s protocol. SCr and urea concentrations were measured pre-operatively and on a daily basis after surgery. Lactacidemia was measured every 6 h, and both urine output and arterial pressure were recorded hourly. A total of 1176 urine samples were collected within 48 h of surgery, with a median of 6 measurements per patient. Samples were centrifuged, then aliquoted, stored at −80 °C and analysed using the ARCHITECT C16000 platform (Abbott Diagnostics, Abbott Park, IL, USA). The first results were reported previously [11], and they showed excellent predictive ability of the urine creatinine normalized ratio of neutrophil gelatinase-associated lipocalin (NGAL) measured within 24 h of surgery for the composite outcome of dialysis and/or death. In the present study, we investigated the complex relationships between AKI (modelled as a latent variable), risk factors of AKI, traditional biomarkers of AKI, uNGAL and post-operative outcomes.

### Statistical analyses

Repeated measurements were analysed using the area under the curve (AUC) calculated by using the trapezoidal method, accounting for the magnitude and the duration of the parameter variation and adjusted for monitoring duration. Urinary excretion of NGAL within the first 12 h after surgery was analysed using the AUCs for absolute concentration, excretion rate and urine creatinine normalized concentration. The metric for the variations of arterial pressure within 24 h of surgery was the AUC below the first quintile per age group (<2 days, 3–28 days or older). SCr was analysed using either the AUCs for the post-operative variation relative to baseline or the AUC for a >50 % post-operative increase relative to baseline. Because the literature is conflicting with regard to the definition of oliguria following cardiac surgery in patients <1 year of age [4, 7, 18], the metric for oliguria was the AUC below the most popular thresholds of urine output (i.e., <0.5, <1 and <2 ml kg^−1^ within 24 h of surgery). Finally, the AKI stage was estimated according to the classification of the Acute Kidney Injury Network (AKIN) [19]. The vasoactive-inotropic score [20], the sum of diuretic dosages administered within 24 h of surgery, and the sum of all blood products used intra-operatively and during the day of surgery (day 0) were also calculated. Fluid balance was computed within 24 h of surgery. A composite variable was adopted to summarize the pre-operative medical history, with a score of 1 attributed for each of the following: need for resuscitation, mechanical ventilation or inotropic support; pre-operative infection; enterocolitis; pulmonary hypertension; preoperative use applies to "diuretics, angiotensin-converting enzyme inhibitors, aminoglycoside antibiotics, vancomycin or intravenous contrast"; or presence of an identified genetic syndrome.

### Data modelling

The dataset contained a large number of correlated variables, and we assumed that they might be a reflection of a limited number of pathophysiological processes. To reduce the number of variables and to identify the potential factors (pathophysiological processes and their surrogates) across the dataset, we used exploratory factor analysis (EFA) with varimax rotation [15]. The latent root criterion with eigenvalues >1 was used to identify the number of potential factors, and the scree test criterion was used to reduce the number of factors. By using EFA, we estimated the loading of each variable on each factor (equivalent to the correlation coefficient between the variable and the factor), as well as the communalities (summary statistic showing how much of the variable’s variance is accounted for by the factor solution). During the EFA refinement process, the variables with low loading (<0.400 for a sample size of 200) and/or low communality (<50) and/or cross-loading were excluded. In cases where variables represented different expressions of the same phenomena (the three NGAL metrics, the three oliguria metrics, the two variation of serum creatinine relative to baseline [ΔsCr] metrics and the AKIN stage, and the systolic and mean arterial pressure variations), the variable with the highest factor loading was used as a surrogate representative. All of the factors retained in the EFA were used to model latent variables.

Next, an SEM was specified using the selected variables [16]. The path coefficient values correspond to the standardized solution of the model and, as such, allow for a direct comparison between the strength of the paths in the model. The amount of the variance shared by two variables corresponds to the square of the numerical value of the path connecting them. The Lagrange multiplier was used to respecify the initial model, and the Bayesian information criterion (BIC) was adopted for refinement of the model specification. The model was evaluated using the root mean square error of approximation (RMSEA), the normed fit index (NFI) and the goodness-of-fit index (GFI). The χ^2^ exact test of fit was not used, since it is too sensitive when applied to large datasets. Individual path coefficients in the model were tested for statistical significance, and 95 % 95 % CIs of all parameters were estimated by bootstrapping with 200 resamples. In the comparison of the path coefficients, we employed the estimation of the 95 % CIs of their difference by bootstrapping with 200 resamples. SEM was performed using the “sem” package, and the basic package of the R software version 2.10.1 for Windows (https://cran.r-project.org/bin/windows/base/old/2.10.1/) was used for all other analyses.

## Results

The characteristics of the study population have been described extensively elsewhere [11], and they are summarized in Table 1. Extracorporeal membrane oxygenation was the only mechanical assist method and peritoneal dialysis the only renal replacement therapy used in the study population.

**Table 1:** Characteristics of the study population

Characteristics	Data
Demographic characteristics	
Age, days	95.8 ± 91.1
≤ 28 days of birth	75 (37.5)
≤ 48 h of birth	5 (2.5)
Weight, kg	4.4 ± 1.5
Medical history before surgery	
Genetic syndrome	8 (4)
Resternotomy	18 (9)
Cardiopulmonary resuscitation	2 (1)
Mechanical ventilation	11 (5.5)
Inotropic support	3 (1.5)
Infection	19 (9.5)
Enteropathy	4 (2)
Medication before surgery	
Furosemide	42 (21)
Spironolactone	12 (6)
Angiotensin-converting enzyme inhibitors	28 (14)
Beta blockers	12 (6)
Prostaglandin E_1_	44 (22)
Aminoglycoside antibiotics	31 (15.5)
Vancomycin	18 (9)
Angiography within 1 week before surgery	22 (11)
Intra-operative variables	
STS-EACTS Congenital Heart Surgery Mortality Score	0.94 ± 0.79
Duration of cardiopulmonary bypass, minutes	118.2 ± 63.0
Surgery requiring aortic cross-clamping	190 (95)
Duration of aortic cross-clamping, minutes	62.9 ± 33.9
Ultrafiltration rate, ml kg^−1^ min^−1^ of bypass	1.4 ± 0.6
Surgery requiring deep hypothermic circulatory arrest	18 (9)
Packed red blood cell transfusions, ml	469.9 ± 153.9
Fresh frozen plasma transfusions, ml	221.1 ± 116.4
Platelet transfusions, ml	61.8 ± 32.7
Post-operative variables	
Requirement for delayed sternal closure	38 (19)
Requirement for extracorporeal membrane oxygenation	3 (1.5)
Peak lactacidemia within 6 h of surgery, mmol L^−1^	3.8 ± 2.0
Peak lactacidemia within 48 h of surgery, mmol L^−1^	3.7 ± 1.9
Vasoactive-inotropic score within 48 h of surgery, μg kg^−1^ min^−1^	15.05 ± 7.29
Delay to sternal closure, days	3.9 ± 3.5
Post-operative renal variables	
Peak serum creatinine within 48 h of surgery, mmol L^−1^)	49.2 ± 23.1
Peak ΔsCr within 48 h of surgery, %	26.4 ± 47.1
Mean urine output within 24 h of surgery, ml kg^−1^ h^−1^	4.1 ± 2.7
Negative fluid balance, ml kg^−1^ 24 h^−1^	−28.06 ± 26.8
Peritoneal dialysis	16 (8)
Duration of peritoneal dialysis, days	0.24 ± 0.96
AKI stage according to AKIN system	
Stage 1	16 (8)
Stage 2	8 (4)
Stage 3	17 (8.5)
Duration of mechanical ventilation, days	1 [0–5]
Duration of intensive care unit stay, days	4.5 [2–7]
In-hospital death	8 (4)

*AKI* acute kidney injury, *AKIN* Acute Kidney Injury Network, *ΔsCr* serum creatinine variation relative to baseline, *STS-EACTS* Society of Thoracic Surgeons and European Association for Cardio-Thoracic Surgery

Data are shown as mean ± SD, median [IQR] or number and proportion

All of the variables used for EFA were metric measurements, except for in-hospital mortality. Both the Bartlett test of sphericity (χ^2^ = 4746 with 406 degrees of freedom, *p* < 0.001) and the Kaiser-Meyer-Olkin measure of sample adequacy (0.804, represents the numerical value of thet result of the Kaiser-Meyer-Olkin test) indicated that the correlations between variables were greater than would be expected by chance, and that the dataset was suitable for EFA. The latent root criterion suggested that the first seven factors would qualify; nevertheless, since only one variable loaded significantly on the seventh factor, the six-factor solution was chosen. The initial factor solution is shown in Additional file 1. The EFA refinement process resulted in a four-factor solution, shown in Table 2 along with the factors and loading values (±0.200 or greater). Together, the four factors explained 61.0 % of the variance in the dataset, a proportion considered satisfactory [15]. Factor 1 explained 19.8 % of the total variance and was related to the outcome variables, which were used to model the Outcome latent variable. Factor 2 explained 18.3 % of the total variance and was represented by the CPB parameter variables, which were used to model the CPB latent construct. Factors 3 and 4 explained, respectively, another 15.8 % and 7.1 % of the total variance and were related to the post-operative hemodynamic parameters, which were used to model the low cardiac output syndrome (LCOS) latent variable, and to the renal parameters, which were used to model the AKI latent variable.

**Table 2:** Exploratory factor analysis with varimax rotation: results of the 4-factor solution

Variable	Factor 1	Factor 2	Factor 3	Factor 4
	Outcome	CPB	LCOS	AKI
Age, days			**−0.773**	
Duration of cardiopulmonary bypass, minutes	0.305	**0.907**		0.242
Duration of aortic cross-clamping, minutes		**0.817**		
Conventional ultrafiltration on bypass, ml		**0.695**		
Blood transfusions on day 0, ml	0.251	0.215	**0.627**	
Lactacidemia, AUC		0.280	**0.680**	
Systolic arterial pressure, AUC^a^	0.209		**0.610**	0.214
Urine output <0.5 ml kg^−1^ h^−1^, AUC	0.188			**0.420**
Increase in serum creatinine >50 % relative to baseline, AUC				**0.538**
Urine creatinine normalized NGAL, AUC^b^		0.393		**0.494**
Duration of mechanical ventilation, days	**0.946**	0.202	0.227	
Length of intensive care unit stay, days	**0.910**		0.227	
In-hospital mortality	**0.613**			0.236
Eigenvalue	5.11	1.71	1.55	1.01
Proportion of the explained variance	0.198	0.183	0.158	0.071

*NGAL* neutrophil gelatinase-associated lipocalin, *AKI* acute kidney injury, *CPB* cardiopulmonary bypass, *LCOS* low cardiac output syndrome

The table shows the estimated factor loadings. The factor loading value is equivalent to the correlation coefficient between the variable and the factor. Only factor loading values greater than ±0.200 are shown. Factor loading values greater than ±0.400 (considered significant for a sample size of 200) are shown in boldface type; they allowed for identification of the variables further used to model the latent constructs Outcome, CPB, LCOS and AKI. Together, the four factors explained 0.610 of the total variance in the dataset, a proportion considered acceptable. AUC accounts for the magnitude and the duration of the parameter variation

^a^Within 24 h of surgery

^b^Within 12 h of surgery. All the other parameters were monitored within 48 h of surgery

**Fig. 1:**
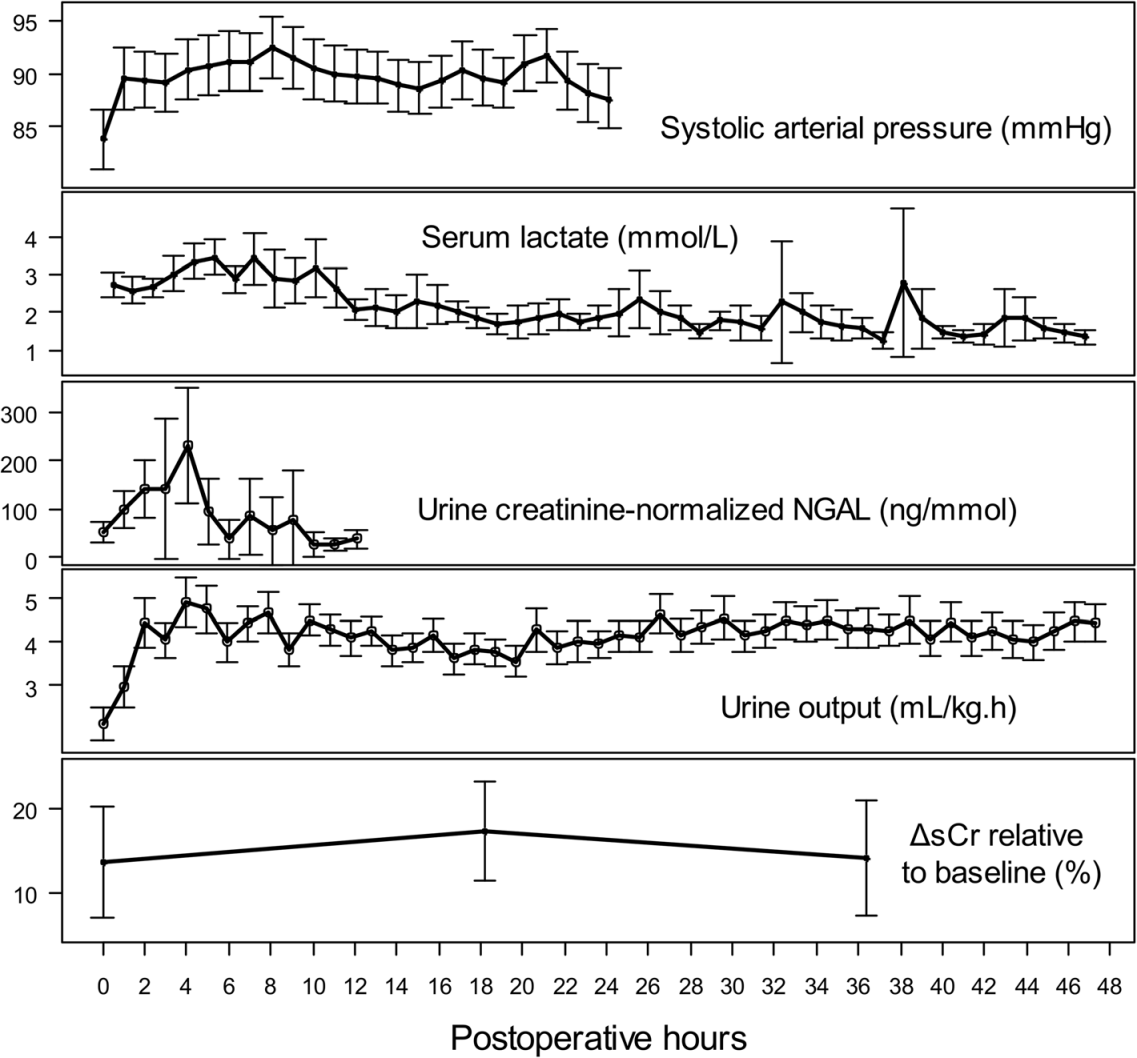
Variation as a function of time for the parameters included in the structural equation model. Duration of monitoring varied between 12 and 48 h after surgery. *NGAL* neutrophil gelatinase-associated lipocalin, *ΔsCr* variation of serum creatinine relative to baseline

**Fig. 2:**
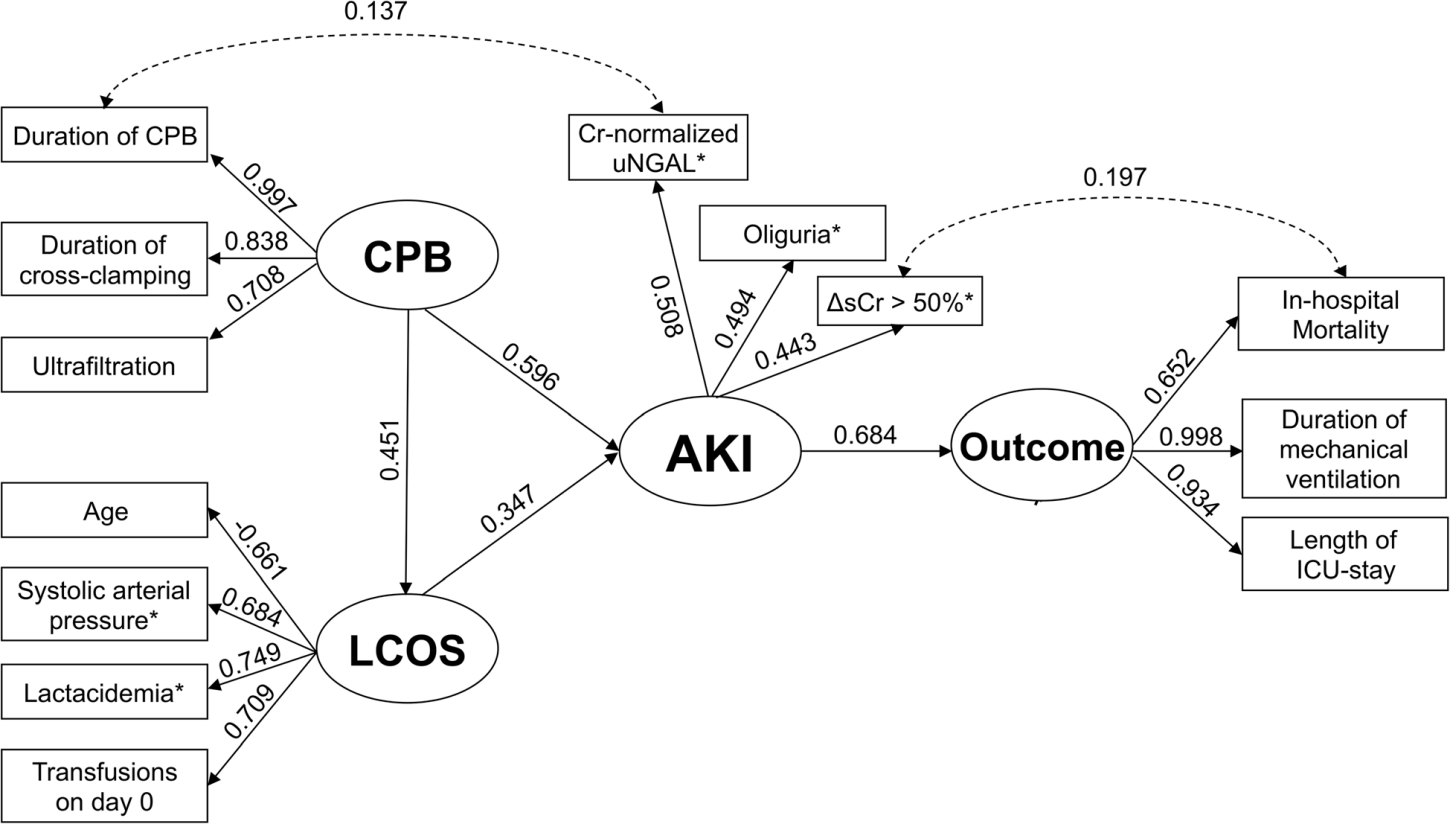
Diagram of the structural equation model. The path coefficients shown above each single-headed arrow are standardized partial regression coefficients, indicating to what extent a change of the variable at the tail of the arrow is transmitted to the variable at the head of the arrow (with all other variables indicated in the diagram held constant). The coefficients shown above double-headed arrows are covariance coefficients. Residual arrows were omitted. All path coefficients were statistically significant (*p* < 0.001). *Modelled using the AUCs, accounting for the magnitude and the duration of the parameter variation. *AKI* acute kidney injury, *CPB* cardiopulmonary bypass, *ICU* intensive care unit, *LCOS* low cardiac output syndrome, *ΔsCr* variation of serum creatinine relative to baseline, *uNGAL* urine neutrophil gelatinase-associated lipocalin

Variation of all of the observed parameters included in the SEM is shown in Fig. 1 as a function of time. Two covariance paths were added during the model refinement process. The first was between creatinine normalized uNGAL and the duration of CPB, and the second was between ΔsCr >50 % and in-hospital mortality. The diagram of the final SEM is shown in Fig. 2 and follows the convention of representing latent variables with ovals and observed variables with rectangles. The path coefficients shown above each single-headed arrow are standardized partial regression coefficients, and the coefficients shown above the double-headed arrows are covariance coefficients. Residual arrows were omitted. All direct path coefficients shown in Table 3 were statistically significant. The RMSEA was 0.085, indicating a reasonable error of approximation of the covariance matrix [21]. The NFI was 0.911 and the GFI was 0.909, indicating good adjustment. The BIC was −167.12.

**Table 3:** Path coefficients of the structural equation model

Direct path of the SEM	Standardized coefficient	95 % CI
CPB → duration of CPB	0.997	0.946–1.051
CPB → duration of cross-clamping	0.838	0.753–0.893
CPB → conventional ultrafiltration	0.708	0.592 to −0.791
LCOS → age	−0.661	−0.762 to −0.559
LCOS → systolic arterial pressure	0.684	0.541–0.791
LCOS → lactacidemia	0.749	0.674–0.822
LCOS → transfusions on day 0	0.709	0.584–0.796
AKI → creatinine normalized uNGAL	0.508	0.202–0.745
AKI → ΔsCr >50 %	0.443	0.273–0.596
AKI → oliguria	0.494	0.250–0.864
Outcome → duration of mechanical ventilation	0.998	0.958–1.043
Outcome → length of ICU stay	0.934	0.905–0.967
Outcome → in-hospital mortality	0.652	0.375–0.784
CPB → AKI	0.596	0.341–0.747
CPB → LCOS	0.451	0.319–0.562
LCOS → AKI	0.347	0.096–0.553
AKI → outcome	0.684	0.532–0.878
Duration of CPB ↔ creatinine normalized uNGAL	0.137	0.077–0.404
In-hospital mortality ↔ ΔsCr >50 %	0.197	0.109–0.271

*AKI* acute kidney injury, *CPB* cardiopulmonary bypass, *Cr* creatinine, *ΔsCr* serum creatinine variation relative to baseline, *LCOS* Low cardiac output syndrome, *ICU* intensive care unit, *uNGAL* urine neutrophil gelatinase-associated lipocalin, *SEM* structural equation modelling

Only the direct path coefficients are shown. The 95 % CIs were estimated by bootstrapping with 200 resamples. All *p* values were <0.001. The values of the compound path coefficients are given in the main text, together with their 95 % CIs

Two common rules in path models are that the numerical value of a compound path is equal to the product of the values of its constituent arrows, and that the correlations between two variables can be expressed as a sum of all direct and/or indirect paths connecting them. Accordingly, the SEM indicated that there was a significantly stronger relationship between CPB and AKI than between LCOS and AKI, with direct/compound path coefficients of 0.820 (i.e., 0.596 + 0.451 × 0.347 + 0.997 × 0.137 × 0.508) with 95 % CI 0.527–0.979 and 0.347 with 95 % CI 0.096–0.553, respectively. The 95 % CI of their difference was 0.062–0.607, which was statistically significant. The SEM also indicated that there was a stronger relationship between AKI and the creatinine normalized uNGAL than between AKI and ΔsCr >50 %, with direct/compound path coefficients of 0.611 (i.e., 0.508 + 0.137 × 0.997 × 0.596 + 0.137 × 0.997 × 0.451 × 0.347) with 95 % CI 0.347–0.777 and 0.443 with 95 % CI 0.273–0.596, respectively. The relationship between AKI and Oliguria was intermediate, with a direct path coefficient of 0.494 with 95 % CI 0.250–0.864. There was a strong relationship between AKI and Outcome, with a path compound coefficient of 0.741 (i.e., 0.684 + 0.443 × 0.197 × 0.652) with 95 % CI 0.610–0.988. There was a non-significant relationship between age and AKI, as suggested by the compound path coefficient of −0.229 (i.e., 0.661 × 0.347) with 95 % CI −0.319 to 0.060.

## Discussion

When modelled as a latent variable, cardiac surgery-related AKI in patients <1 year of age appeared to be a consequence of tubular injury, predominately mediated by the CPB, and strongly related to early NGAL excretion. The strong relationship with the outcome after surgery suggests that tubular injury is the most relevant process to monitor in this setting. The present findings also suggest that urine creatinine normalized NGAL concentration is a more accurate marker of AKI than ΔsCr in this setting, and that oliguria is a marker of intermediate value. According to the present results, age at surgery has little impact on the occurrence of post-operative AKI within the first year of life.

AKI following cardiac surgery is known to be multi-factorial, and several pre-, intra- and post-operative factors have been reported to increase the risk of post-operative AKI [22]. Low systemic oxygen delivery and low blood pressure have recently been pointed out as main drivers for severe AKI in this context [23]. Specific risk factors based on physiological features related to age and due to specific surgical requirements have been reported in very young patients. Because of the small sample size of the present study, not all of the known and/or suspected risk factors for post-operative AKI were covered here. To limit the number of confounding variables, the present analysis was focused on a homogeneous group of patients <1 year of age.

Even though it is more commonly adopted in the social sciences, SEM has found a few applications in the field of nephrology, such as in assessment of the relationship between chronic kidney injury and periodontal disease [24] and in analysis of the diagnostic accuracy of AKI biomarkers [25]. SEM was chosen for the present study because it appeared to be well-suited for investigation of the pathophysiological pathways whereby cardiac surgery-related AKI develops. Since the model showed a close fit, the pathways considered here are statistically plausible. Also, by providing numerical estimates for the parameters, SEM allows for an estimation of the strength of the relationships. Both length of CPB and LCOS have been acknowledged to be major risk factors for AKI in paediatric studies [2, 4–8]. The new finding in the present study is the attribution of the leading role to CPB. With a path coefficient of 0.820, 67.2 % of the variability of the risk of AKI was explained by the CPB, whereas with a path coefficient of 0.347, only 12.0 % of the variability in the occurrence of post-operative LCOS translated into AKI risk. A parallel can easily be drawn with the controversy around off-pump coronary artery bypass in adults: off-pump surgery removes the bypass circuit but can be associated with greater hemodynamic instability as the heart is manipulated to access the coronary arteries. Such situation allows separation of the risk factors specifically associated with the CPB itself from pre-, intra- and post-operative factors. Despite ongoing controversy, data support a lower risk for AKI in patients who undergo off-pump surgery [3]. It is largely assumed that oxidative stress, inflammation and ischemia are the most prominent mechanisms of cardiac surgery-related AKI [22]. They could all be caused by CPB because CPB is associated with the generation of free haemoglobin and iron through haemolysis [3] and contributes to oxidative stress [26]. CPB also causes systemic inflammatory response syndrome [27], and it is not known whether renal autoregulation is maintained and adequate perfusion is provided to the kidney during non-pulsatile CPB in children.

In accordance with previous literature showing that uNGAL can reveal AKI in infants who undergo cardiac surgery [11–13] and with the results of the EFA, uNGAL was used in the present study to model the AKI latent variable. The SEM suggested that the relationship between AKI and uNGAL was stronger than that between AKI and ΔsCr. The dissociation between the increase in sCr and uNGAL excretion in patients susceptible to AKI is not a new finding, and it is acknowledged that uNGAL and sCr provide signals of a different nature. In contrast to conventional markers such as sCr and urea, uNGAL reflects not kidney function but structural damage of the tubular cells [28]. Haase et al. conducted a pooled analysis including more than 2000 patients with cardiorenal syndrome [29]. Patients with positive uNGAL and sCr criteria of AKI had the worst outcomes. Nevertheless, the authors identified a subgroup of NGAL^+^/sCr^−^ patients diagnosed with AKI by means of NGAL, which would have been diagnosed as non-AKI using sCr criteria. These patients had tubular injury without evidence of glomerular function deficit. The probability of renal replacement therapy increased by more than 16-fold in NGAL^+^/sCr^−^ patients as compared with NGAL^−^/sCr^−^ patients, length of stay was >70 % longer and hospital mortality doubled. A smaller group of patients were NGAL^−^/sCr^+^. They exhibited pre-renal azotaemia, implying loss of renal function without evidence of tubular injury, and their outcome was found to be intermediate in severity. Such results suggest that tubular injury is a more clinically relevant event than isolated functional deficit. It is likely that the underlying pathological mechanism identified by the AKI construct in the present study was for the main part tubular injury with subsequent tubular expression and excretion of NGAL. This hypothesis is further strengthened by the direct covariance path added between uNGAL and the duration of CPB, which improved the adequacy of the SEM. However, there was a strong relationship between the AKI construct and the outcome, with a compound coefficient of 0.741, translating to a 54.9 % explanation of the variability in the post-operative outcome. This is in accordance with previous literature on infants [1, 2] and strengthens the validity of our AKI construct. Together, the present findings suggest that the most prominent pathophysiological process and the most clinically relevant event in cardiac surgery-related AKI is tubular injury.

Nevertheless, our findings do not disqualify functional glomerular deficit as a pathophysiological process involved in cardiac surgery-related AKI. Moreover, the addition of a direct covariance path between ΔsCr >50 % and in-hospital mortality contributed to the refinement of the SEM. sCr usually decreases following CPB in infants, and infants with AKI may have a decreased sCr post-operatively [10]. Indeed, a 50 % sCr increase, especially when it occurs within the first 48 h of surgery, is likely to be the result of a severe injury to the kidney. Experimental AKI models support the hypothesis that tubular injury precedes sustained severe GFRimpairement [30], and it is likely that, here the ΔsCr >50 % criterion identified infants with an advanced stage of tubular injury. Our findings are therefore in accordance with previous studies of infants showing that only large increases in sCr affect survival [1, 2]. Furthermore, they highlight the importance of monitoring markers of tubular injury to identify patients in the early stage of the disease.

Our study was conducted in a population of cardiac surgery neonates and young infants, a population never previously covered in large studies [31]. Younger age has traditionally been associated with an increased risk of post-operative AKI, due to the relative renal immaturity of the neonate [2, 32, 33]. Within the first days after birth, the physiologically very low GFR, together with the rapid changes in the distribution of water between extracellular and intracellular fluid compartments, makes it difficult for the neonate’s system to control water balance, especially if kidney function is further impaired following CPB. In a recent study of infants, researchers found a 3 % decrease in the risk of cardiac surgery-related AKI per day following birth [2]. Consequently, many institutional protocols advocate supportive care rather than corrective surgery in the early neonatal period in order to allow for weight gain and organ maturation. However, this is controversial because an absence of association between body weight and short-term outcomes following paediatric cardiac surgery has also been reported [34]. Moreover, recent data show that the risk of cerebral damage in neonates with cyanotic heart disease increases with the time to corrective surgery [35]. Under normal conditions, the partial pressure of oxygen in arterial blood (PO_2_) in the inner medulla is 20 mmHg [36]. Most infants requiring surgery early in life have cyanotic heart diseases. PO_2_ is as low as 40 mmHg in patients with cyanotic cardiac disease, and it is likely that the PO_2_ in the inner medulla becomes critically low and that the risk due to renal immaturity is compounded by the risk of tubular hypoxia. In the present study, we found the relationship between age and risk of AKI to be very weak, with a compound path coefficient of only −0.229, translating to only a 5.2 % explanation of the AKI risk. With regard to the risk of post-operative AKI, our findings suggest that there is no benefit of delaying surgery in neonates. Conversely, we found a strong association between age and the occurrence of LCOS, which is in accordance with published data [37, 38].

### Limitations

The major limitation of this observational study is the inability to infer any causality, regardless of how well the SEM fits with the data. The small sample size did not allow for analysis of all of the possible risk factors for AKI, and our interpretation is consequently vulnerable to any important variable that could have been omitted. The weak relationship between LCOS and AKI could have been partly due to the time offset between the monitoring of the parameters used to model the two latent variables.

## Conclusions

The findings in the present study suggest that cardiac surgery-related AKI in patients <1 year of age is a consequence of tubular injury, predominately driven by the CPB, and strongly related to early NGAL excretion. Our findings suggest that urine creatinine normalized NGAL concentration is a more accurate marker of AKI than sCr variations in this setting, and that oliguria is a marker of intermediate value. They also suggest that age has little impact on the occurrence of post-operative AKI within the first year of life.

## Key messages

- Cardiac surgery-related AKI in patients <1 year of age is a consequence of tubular injury.
- The most prominent risk factor for post-operative AKI is the duration of the cardiopulmonary bypass.
- Urine creatinine normalized NGAL concentration is a more accurate marker of AKI than creatinine variations.
- Age at surgery has little impact on the occurrence of post-operative AKI within the first year of life.

## Abbreviations

AKI, acute kidney injury; AKIN, Acute Kidney Injury Network; BIC, Bayesian information criterion; CPB, cardiopulmonary bypass; EFA, exploratory factor analysis; GFI, goodness-of-fit index; GFR, glomerular filtration rate; ICU, intensive care unit; LCOS, low cardiac output syndrome; NFI, normed fit index; NGAL, neutrophil gelatinase-associated lipocalin; PO2, partial pressure of oxygen in arterial blood; RMSEA, root mean square error of approximation; sCr, serum creatinine concentration; ΔsCr, variation of serum creatinine relative to baseline; SEM, structural equation modelling; STS-EACTS, Society of Thoracic Surgeons and European Association for Cardio-Thoracic Surgery; uNGAL, urine neutrophil gelatinase-associated lipocalin

## Additional file

Additional file 1: Exploratory factor analysis with varimax rotation: results of the initial model, including all of the available variables. (DOCX 16 kb)
