# Supplementary material for: Structural equation modelling exploration of the key pathophysiological processes involved in cardiac surgery-related acute kidney injury in infants
Source: Crit Care. 2016 Jun 5;20:171. doi: 10.1186/s13054-016-1350-1 (PMC4893417; doi:10.1186/s13054-016-1350-1)
Supplement: Additional file 1: — Exploratory factor analysis with varimax rotation: results of the initial model, including all of the available variables. (DOCX 16 kb) [file 13054_2016_1350_MOESM1_ESM.docx]

**Additional file 1**. **Exploratory factor analysis with varimax rotation: results of the initial model, including all of the available variables.**

The Bartlett test of sphericity (a χ2 of ~~4960~~ 4746 with 406 degrees of freedom, p<0.001) and the Kaiser-Meyer-Olkin measure of sample adequacy (of ~~0.794~~ 0.804) indicated that the dataset was suitable for exploratory factor analysis. The table shows the estimated factor loadings and each variable’s communality.

The factor loading value is equivalent to the correlation coefficient between the variable and the factor. Communalities are summary statistics showing how much variance of a particular variable is accounted for by the factor solution. Only factor loadings larger than ± 0.200 are shown. The variables with factor loadings above 0.400 (considered significant for a sample size of 200) [15], and with communalities above 50, sufficient to be retained as candidates for further analysis [15], are shown in bold.

| Variable | Factor 1 | Factor 2 | Factor 3 | Factor 4 | Factor 5 | Factor 6 | Communality |
| --- | --- | --- | --- | --- | --- | --- | --- |
| **Age (days)**  Preoperative score  **Duration of cardiopulmonary bypass (min)**  **Duration of ortic cross-clamping (min)**  Deep hypothermic circulatory arrest (min)  STS-EACTS Congenital Heart Surgery Mortality score  **Conventional ultrafiltration on bypass (mL)**  **Blood transfusions on day 0 (mL)**  Sternum left open (days)  Vasoactive-inotrope score  **Lactacidemia (AUC)**  **Systolic arterial pressure (AUC)***  Mean arterial pressure (AUC)*  Days on extracorporeal membrane oxygenation  Furosemid (mg kg^-1^ day^-1^)  **Urine output < 0.5mL kg^-1^ h^-1^ (AUC)**  **Urine output < 1mL kg^-1^ h^-1^ (AUC)**  Urine output < 2mL kg^-1^ h^-1^ (AUC)  Fluid balance on the operative day (mL kg^-1^ day^-1^)  **Increase in serum creatinine > 50% relative to baseline (AUC)**  **Serum creatinine (AUC)**  Serum urea (AUC)  **Urine creatinine-normalzed NGAL (AUC)****  **Urine NGAL absolute concentration (AUC)****  **Urine NGAL absolute excretion (AUC)****  Duration of peritoneal dialysis (days)  **Stage of kidney injury according to AKIN**  **Duration of mechanical ventilation (days)**  **Length of Intensive Care Unit stay (days)**  **In-hospital mortality** | **0.872**  **0.823**  0.386  0.456  **0.747**  0.305  0.412  0.369  0.340  0.217  0.203  0.285  0.220  0.321  0.326  0.297  0.259  0.227  0.262  0.232 | 0.226  0.204  0.349  0.256  0.250  0.281  0.312  0.239  0.313  0.395  0.349  **0.917**  **0.874**  **0.657** | **-0.880**  0.240  **0.586**  0.227  **0.548**  **0.506**  0.470  0.224 | 0.362  0.200  0.530  0.278  0.269  **0.899**  **0.871**  0.350  0.261  0.230 **0.525**  0.251 | 0.265  0.279  0.281  0.367  **0.920**  **0.748**  **0.864** | 0.298  0.298  **0.908**  **0.871**  0.203  0.393  0.262  0.221 | 77.67  9.16  93.32  73.43  21.50  28.66  59.29  50.36  39.79  24.32  56.80  57.07  47.47  47.42  33.16  99.50  95.68  2.38  12.63  85.86  86.73  37.86  76.96  99.50  83.78  42.14  56.85  99.50  91.87  54.13 |
| Eigenvalue | 9.98 | 3.05 | 2.72 | 1.87 | 1.83 | 1.37 |  |
| Proportion of the explained variance | 0.118 | 0.101 | 0.101 | 0.093 | 0.089 | 0.077 |  |

*within 24 hours of surgery, ** within 12 hours of surgery. All the other parameters were monitored within 48 hours of surgery.

AUC: area under the curve (accounting for the magnitude and the duration of the parameter variation); NGAL: Neutrophil Gelatinase-Associated Lipocaline;
